# Conference Calendar

**DOI:** 10.1002/cfg.346

**Published:** 2003-12


					Comparative and Functional Genomics
Comp Funct Genom 2003; 4: 678-680.
Published online in Wiley InterScience (www.interscience.wiley.com). DOI: 10.1002/cfg.346
Conference Calendar
March-May 2004
Animal models
45th Annual Drosophila Research Conference,
March 24-28
http://flybase.bio.indiana.edu/docs/news/
announcements/meetings/
Bacteria
ASM -- Genomes 2004: International
Conference on Analysis of Microbial and Other
Genomes, April 14-17
http://www.pasteur.fr/recherche/unites/gmp/
sitegmp/gmp conf.html
Bioinformatics
Computational and Systems Neuroscience,
March 24-28
http://meetings.cshl.org/2004/2004cosyne.htm
8th Annual International Conference on
Research in Computational Molecular Biology
(RECOMB 2004), March 27-31
http://recomb04.sdsc.edu/
Basel Computational Biology Conference 2003:
Life Sciences Meet IT, April 3-4
http://www.bc2.ch/2003/index.html
Workshop on Genomic Signal Processing and
Statistics (GENSIPS), May 26-28
http://www.cis.jhu.edu/gensips2004/
Evolution/comparative genomics
CSHL -- Evolution of Developmental
Diversity, March 31-April 4
http://meetings.cshl.org/2004/2004divers.htm
Functional genomics
KEY -- siRNAs and miRNAs, April 14-19
http://www.keystonesymposia.org/Meetings/
ViewMeetings.cfm?MeetingID=701
CHI -- Phage Display Technologies, April
26-27
http://www.healthtech.com/2004/pgd/index.asp
RNAi-2004-Boston Meeting: RNA Interference:
Biology to Drugs and Therapeutics, May 2-4
http://www.expressgenes.com/rnai2004.htm
8th World Congress on Biosensors, May
24-26
http://www.biosensors-congress.com/
General
Nanotech 2004, March 7-11
http://www.nanotech2004.com/
KEY -- Biological Discovery Using Diverse
High-throughput Data, March 30-April 4
http://www.keystonesymposia.org/Meetings/
ViewMeetings.cfm?MeetingID=669
CHI -- Genomic and Proteomic Sample
Preparation, April 26-27
http://www.healthtech.com/2004/smp/index.asp
CSHL -- The Biology of Genomes, May 12-16
http://meetings.cshl.org/2004/2004genome.htm
Human genome
HUGO -- Human Genome Meeting
(HGM2004), April 4-7
http://hgm2004.hgu.mrc.ac.uk/
Copyright  2003 John Wiley & Sons, Ltd.
Conference Calendar 679
Pathways and metabolomics
ASM -- Integrating Metabolism and Genomics
(IMAGE), April 30-May 3
http://www.asm.org/Meetings/index.asp?
bid=18526
Pharmacogenomics
Applications of Molecular Biology in Modern
Medicine, March 6-11
http://hsccwww.kuniv.edu.kw/conference2003/
conference2003.htm
IBC -- Drug Discovery Technology, March
8-10
http://www.drugdisc.com/europe/
IBC -- Screentech World Summit, March
21-25
http://www.lifesciencesinfo.com/screentech/
CHI -- Molecular Medicine Tri-Conference,
March 23-26
http://www.chiMolecularMed.com
CHI -- Microarrays in Medicine, April 26-27
http://www.healthtech.com/2004/mar/index.asp
CHI -- Glycomics: Carbohydrates in Drug
Development, April 26-27
http://www.healthtech.com/2004/gly/index.asp
IBC -- Tides 2004, April 26-29
http://www.lifesciencesinfo.com/tides
IIR DE -- NanoTrends, May 26-28
http://www.iir-germany.com/nanotrends/
CHI -- Intelligent Drug Discovery and
Development, May 27-30
http://www.chi-intelligentdrug.com/
Plants
KEY -- Comparative Genomics of Plants,
March 4-9
http://www.keystonesymposia.org/Meetings/
ViewMeetings.cfm?MeetingID=688
Crop Functional Genomics, April 7-10
http://www.ispfg.org/
BS -- Post-transcriptional Regulation of Plant
Gene Expression, April 15-18
http://www.biochemistry.org/meetings/
programme.cfm?Meeting No=SA019
Proteomics
ABRF -- Integrating Technologies in
Proteomics and Genomics, February
28-March 2
http://mercury.faseb.org/abrf2004/default.htm
1st Pacific-Rim International Conference on
Protein Science, April 14-18
http://edpex104.bcasj.or.jp/pricps2004/
ASMS -- 51st Annual Conference on Mass
Spectrometry and Allied Topics, May 23-27
http://www.asms.org/
Structural genomics
KEY -- Structural Genomics, April 13-19
http://www.keystonesymposia.org/Meetings/
ViewMeetings.cfm?MeetingID=672
KEY -- Frontiers in Structural Biology, April
13-19
http://www.keystonesymposia.org/Meetings/
ViewMeetings.cfm?MeetingID=673
Systems biology
CSHL -- Systems Biology: Genomic
Approaches to Transcriptional Regulation,
March 4-7
http://meetings.cshl.org/2004/2004systems.htm
Copyright  2003 John Wiley & Sons, Ltd. Comp Funct Genom 2003; 4: 678-680.
680 Conference Calendar
Biological Systems Simulation Conference,
March 8-10
http://conference.ifas.ufl.edu/bssg/index.html
Transcriptomics
1st International qPCR Symposium and
Application Workshop: Transcriptomics,
Clinical Diagnostics and Gene Quantification,
March 3-6
http://www.wzw.tum.de/gene-quantification/
qpcr2004/index.shtml
Yeasts and fungi
ASM -- Candida and Candidiasis, March 18-22
http://www.asm.org/Meetings/index.asp?
bid=17814
Physiology of Yeasts and Filamentous Fungi
(PYFF2), March 24-28
http://pyff2.insa-tlse.fr/
SGM -- 154th Meeting, March 29-April 2
http://www.sgm.ac.uk/meetings/MTGPAGES/
Bath.cfm
Biotech industry
BioSquare 2004 -- Fourth Annual International
Partnering Conference, March 10-12
http://www.ebdgroup.com/biosquare/
Key
ABRF: Association of Biomolecular Resource
Facilities: http://www.abrf.org
ASM: American Society for Microbiology:
http://www.asm.org
ASMS: American Society for Mass Spectrometry:
http://www.asms.org/
BS: Biochemical Society:
http://www.biochemistry.org/
CHI: Cambridge Healthtech Institute:
http://www.healthtech.com
CSHL: Cold Spring Harbor Laboratories:
http://meetings.cshl.org/index.htm
HUGO: Human Genome Organization:
http://www.hugo-international.org/hugo/
IBC: IBC Conferences: http://www.ibc-lifesci.com
IIR DE: IIR Germany: http://www.iir.de
KEY: Keystone Symposia:
http://www.symposia.com/
SGM: Society for General Microbiology:
http://www.sgm.ac.uk
Copyright  2003 John Wiley & Sons, Ltd. Comp Funct Genom 2003; 4: 678-680.

				

